# Ex vivo testing of intact eye globes under inflation conditions to determine regional variation of mechanical stiffness

**DOI:** 10.1186/s40662-016-0052-8

**Published:** 2016-08-10

**Authors:** Charles Whitford, Akram Joda, Steve Jones, Fangjun Bao, Paolo Rama, Ahmed Elsheikh

**Affiliations:** 1 School of Engineering, University of Liverpool, Liverpool, L69 3GH UK; 2 Department of Mechanical Engineering, King Faisal University, Hofuf, Saudi Arabia; 3 Eye Hospital, WenZhou Medical University, WenZhou, China; 4 Ophthalmology Department, San Raffaelle Hospital, Milan, Italy; 5 National Institute for Health Research (NIHR) Biomedical Research Centre at Moorfields Eye Hospital NHS Foundation Trust and UCL Institute of Ophthalmology, London, UK

**Keywords:** Ocular biomechanics, Experimental testing, Digital image correlation

## Abstract

**Background:**

The eye globe exhibits significant regional variation of mechanical behaviour. The aim of this present study is to develop a new experimental technique for testing intact eye globes in a form that is representative of in vivo conditions, and therefore suitable for determining the material properties of the complete outer ocular tunic.

**Methods:**

A test rig has been developed to provide closed-loop control of either applied intra-ocular pressure or resulting apical displacement; measurement of displacements across the external surface of the eye globe using high-resolution digital cameras and digital image correlation software; prevention of rigid-body motion and protection of the ocular surface from environmental drying. The method has been demonstrated on one human and one porcine eye globe, which were cyclically loaded. Finite element models based on specimen specific tomography, free from rotational symmetry, were used along with experimental pressure-displacement data in an inverse analysis process to derive the mechanical properties of tissue in different regions of the eye’s outer tunic.

**Results:**

The test method enabled monitoring of mechanical response to intraocular pressure variation across the surface of the eye globe. For the two eyes tested, the method showed a gradual change in the sclera’s stiffness from a maximum at the limbus to a minimum at the posterior pole, while in the cornea the stiffness was highest at the centre and lowest in the peripheral zone. Further, for both the sclera and cornea, the load–displacement behaviour did not vary significantly between loading cycles.

**Conclusions:**

The first methodology capable of mechanically testing intact eye globes, with applied loads and boundary conditions that closely represent in vivo conditions is introduced. The method enables determination of the regional variation in mechanical behaviour across the ocular surface.

## Background

Simulating the response of the ocular vessel to external and internal forces is of particular interest as it can lead to developments in diagnostic and corrective procedures for various degenerative visual conditions such as glaucoma, keratoconus and myopia [1–6]. Accurate simulations require knowledge of the tissue’s material properties, geometry, loading and boundary conditions, in addition to the numerical tools, with which to describe these details. Simulations can be carried out using numerical tools in the form of finite element models, and the material properties described within these models take the form of constitutive numerical relationships, which can encompass multiple facets of material behaviour.

The multi-faceted behaviour of corneoscleral tissue can be described as anisotropic, hyperelastic [7, 8] and viscoelastic [9, 10]. The stroma is the primary contributor to this behaviour and in the human cornea constitutes 90 % of its thickness. It consists of stacked lamellae of collagen fibrils embedded in a hydrated matrix of proteoglycans, glycoproteins, and keratocytes [11–13]. Similarly, the sclera consists predominantly of collagen fibrils [8, 13] combined as either interlaced bundles or defined lamellae. The fibrils are heterotypic structures as they combine high proportions of collagen types I and III with lower proportions of types V and VI [14, 15]. The straightening of collagen fibrils under stress [16, 17] is a main cause of hyperelastic behaviour where gradual stiffening of tissue under stress continues until most fibres have become taut and able to dominate the behaviour with a linear response to loading. On the other hand, viscoelastic behaviour results from the large fluid component of corneoscleral tissue, which introduces a long-term response to loading and strain-rate dependency. These two observations illustrate the dependence of behaviour on the ocular microstructure, which means that reconfiguration of the microstructure (or re-alignment of the collagen fibres), such as that due to surgical manipulation or changes in the anisotropic strain distribution [9, 18, 19], should be avoided in experimental testing, otherwise the mechanical behaviour would not be relevant to the dominant in vivo conditions.

Several biomechanical studies have been conducted on ex vivo ocular tissues employing mainly uniaxial tension tests [20–26] and inflation tests on separated corneas [27–30] and part scleras [31–33]. Comparative studies of the mechanical stiffness (as measured by the tangent modulus) obtained from uniaxial and inflation tests observed a significant increase in values obtained from the former tests [34, 35]. The higher stiffness estimation was attributed in part to the non-physiologic loading conditions and the higher strain rates commonly employed in uniaxial tension tests [34]. Further, inflation tests of separated corneas or part scleras have a number of drawbacks, namely that (1) the behaviour trends obtained for the corneas and scleras cannot be correlated since the specimens rarely come from the same donor, (2) the anterior sclera is not characterised since it is usually the clamp site for both cornea and sclera specimens, and (3) providing the cornea and sclera specimens with rigid edges during the test creates edge conditions and local stress distributions that are not physiological.

In this study, a case is made for applying the inflation concept on intact eye globes to avoid the shortfalls of both the uniaxial testing of tissue strips and the inflation testing of corneal buttons or scleral cups. While globe inflation testing has been attempted before, the studies were limited to observations of parts of the eye globe; such as posterior sclera [36], anterior segment [37, 38] or around a single horizon [36]. Further, earlier studies have employed boundary conditions that were dissimilar to in vivo conditions and may therefore have affected to the globe’s response to internal pressure loading.

The present study extends behaviour observations to the whole ocular surface, attempts to use more physiologic boundary conditions and illustrates the new method through an initial experimental test programme involving cyclic inflation of intact eye globes. Attention has been given, as much as possible, to ensure consistency with the in vivo conditions of the eye in order to avoid the main sources of error affecting the reliability of the target material properties of the ocular tissue.

In addition to the experimental testing method, the procedure includes a numerical inverse analysis method that uses the experimental pressure-deformation data to determine the tissue’s hyperelastic stress–strain properties across the ocular surface.

## Methods

### Test method

**Fig. 1:**
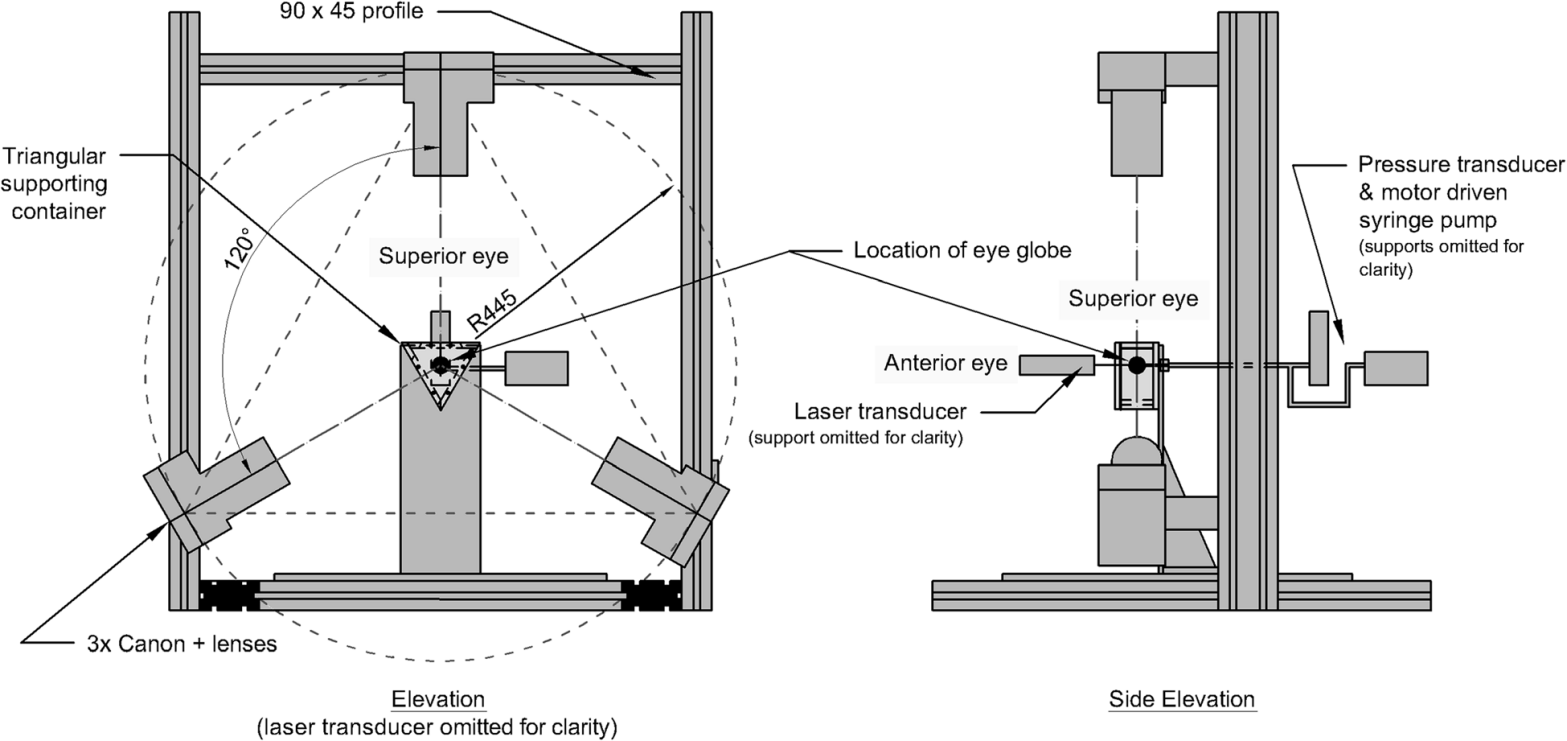
Diagram of the test rig highlighting key components and the arrangement of the measurement equipment in relation to the orientation of the eye globe

Instrumentation has been developed to support, protect and monitor the intact eye globe while allowing simple computer control of test procedure and data collection. A diagram of the test rig is provided in Fig. 1. A fixed borosilicate glass box contains the intact globe and enables its suspension in a clear gelatine material that (1) protects the external surface of the ocular vessel from environmental conditions, (2) provides the eye with a support system, which restricts free-body motion, is more uniform and offers a better representation of physiologic conditions than traditional support systems, and (3) enables an unobstructed view of the entire ocular surface from outside the glass box.

A 2 × 50 mm hypodermic needle was inserted into the ocular cavity through the posterior pole, passed through the back wall of the glass box and its support system and connected through a pipe network to a motor-driven syringe pump, which provided changes in the applied pressure. Fixed at the same elevation as the centre of the eye was a pressure transducer (FDW 060-K262-01, RDP Electronics, USA) that measured the applied pressure with a 0.1 mmHg resolution.

The deformation of the eye that resulted from changing the internal pressure by the syringe pump was measured using a system of three high-resolution, digital cameras (550D, Canon, Japan) with 100 mm (fixed-focal-length) macro lenses that were mounted on a support frame designed for both access and rigidity. The cameras were positioned around the equator of the eye globe facing perpendicular to the sides of the triangular support box. The camera images were initially used to obtain sufficient topography information to construct the eye-specific FE numerical model of the test specimen used in later inverse analysis. The cameras (Fig. 1) were further used during the test to obtain images of the deformed shape of the eye, which were then analysed using digital image correlation (DIC) software (Istra4D, Dantec Dynamics A/S, Denmark) to quantify the displacement distribution across the surface of the eye.

In addition, a laser displacement sensor (LK-2001, Keyence, UK) measured the displacement at the cornea’s apex with 1 μm resolution. This addition to the test rig helped (1) enable the conduct of the test while controlling the displacement at the apex, and (2) provided a direct measurement of an important displacement parameter that aided the validation of the deformation measurements obtained from analysis of the camera images.

### Test control

**Fig. 2:**
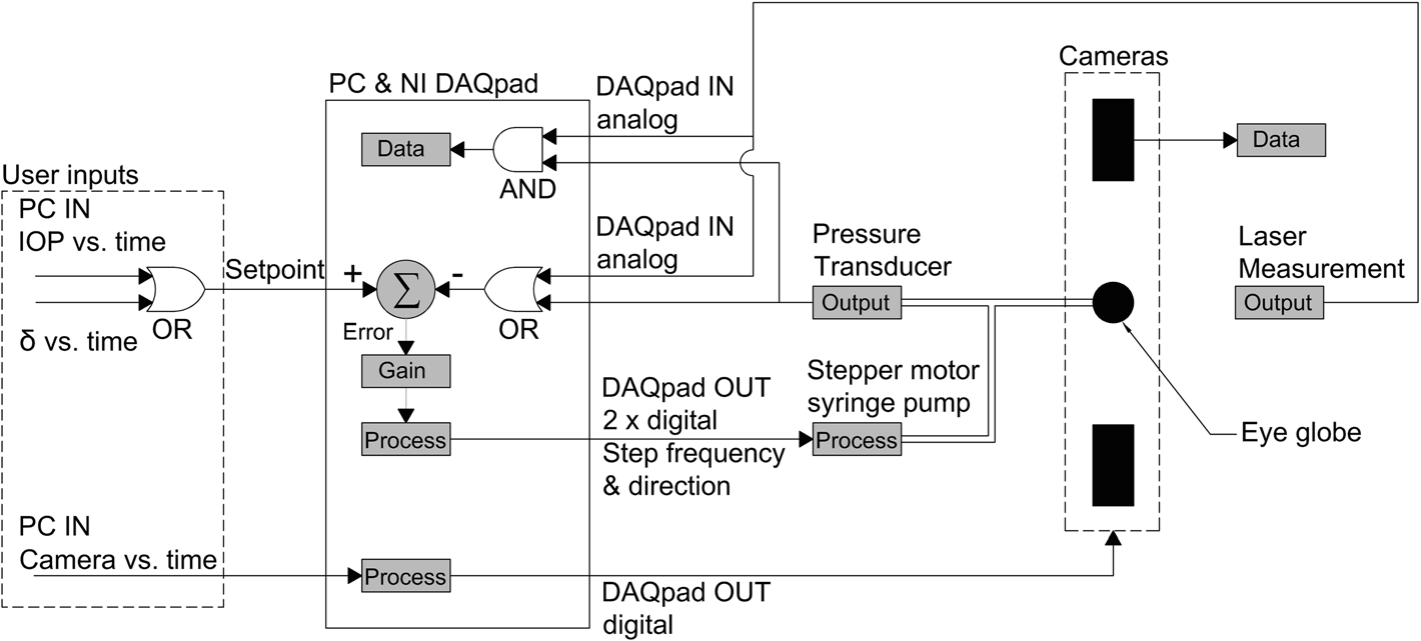
Schematic diagram of the LabVIEW software used to control inflation test

A LabVIEW program has been developed to record data and control the test using a closed-loop system through a data acquisition card (DAQpad 6015, National Instruments, USA). The control and data acquisition system is shown schematically in Fig. 2. The system enables conduct of the test through either control of applied pressure (*p*) or resulting displacement (*δ*) at the corneal apex using a non-linear, proportional-gain, control of the motorised syringe pump. Trials have been conducted to ensure the system provided stable rates of change in pressure (*dp/dt*) or displacement (*dδ/dt*). During the test, applied pressure and displacement at the corneal apex were recorded every 50 ms, and filtered through a 4 Hz low-pass filter, which was assessed to be a suitable sampling rate at the calibration trials. Further, the LabVIEW software triggered all cameras simultaneously at specified times during the test, and the images were collected and used later in the construction of specimen-specific eye models and determining the distribution of deformation across the ocular surface.

### Gelatine support system

The eye specimen was cast into a gelatine support, which was produced from Type-A gelatine flakes derived from porcine skin (Sigma-Aldrich cat. No. G2500). This particular gelatine was chosen from trials for its clarity and low spring stiffness and because it had been reported not to chemically alter collagen based tissue [39]. In order to determine the effect of the gelatine on eye globe deformation, its spring stiffness *k’* was measured using an Instron uniaxial machine fitted with a 10 N load cell and found to be linear of the value *k*^'^ = *F*/(*δA*) = 0.0001 *N*/*mm*^3^, where *F* is the applied force, *δ* the resulting deformation, and *A* the cross-sectional area of gelatine material.

Finite element analysis (FEA) was conducted using the non-linear solver Abaqus/Standard 6.13 (Dassault Systèmes Simulia Corp., Rhode Island, USA) to determine the effect of this stiffness on the deformation of an inflating eye globe. The topography of the model was described by: a corneal radius (7.8 mm) and shape factor (0.82); a sclera radius (11.5 mm); a central corneal thickness, CCT (545 μm – reported as average value in a number of previous studies [40]) and a peripheral corneal thickness (695 μm, which was consistent with Gullstrand’s No. 1 schematic eye [41]). The intraocular void was represented by fluid cavity bound by the internal surface of the ocular vessel to which it provided a uniform hydrostatic pressure that varied in the analysis to inflate the eye globe model from 0–30 mmHg.

Comparison between a control finite element model (FEM) with no gelatine support and a model with an external spring condition matching the measured spring stiffness of the gelatine was conducted. Table 1 provides the deformations and comparison at 30 mmHg. The difference in deformation ranges between 0.05 % and 0.52 %. Cavity volume was also estimated in the analysis, and the internal volume of the spring-supported model was 0.06 mm^3^ (<0.01 %) less than the control model, once both were loaded to 30 mmHg. Based on these results, the spring effect of the gelatine on the experiments was considered negligible.

**Table 1:** Deformation at discrete nodal locations from 0–30 mmHg

	Control FEM (displacement)	FEM with simulation of gelatine support (displacement, difference)
Anterior pole	202 μm	201 μm, 0.49 %
Limbus	69.8 μm	69.5 μm, 0.52 %
Equator	58.2 μm	58.2 μm, −0.05 %
Posterior pole	86.8 μm	86.3 μm, 0.52 %

*FEM* = finite element model

### Calibration of the laser displacement sensor

The laser displacement device uses an optical triangulation position sensor to determine the distance to a near object by the recorded change in angle between the output beam and the reflected beam. Altering the media the laser travels through consequently alters the refractive angle, which in turn affects the triangulation angle. A linear displacement calibrator with a resolution of 1 μm was used to recalibrate the laser displacement transducer through the glass box and gelatine by directly controlling and changing the distance measured by the laser. The relationship between the known relative distance and the relative distance measurements from the laser provided the calibration factor to correct the laser readings. The resulting root mean square (RMS) error after recalibration was 2.7 μm.

### Deformation measurement by digital image correlation

DIC is an established technique that relies on the analysis of successive camera images taken for a test specimen to derive the deformation distribution across the specimen surface [6]. This technique has already been successfully applied, in various forms, to ocular tests [21, 28, 32]. This paper describes the application of two-dimensional DIC to determine the planar deformation of the eye globes. For each camera, we consider the circumferential horizon of the eye to be the planar surface. DIC was performed using the software Istra4D. There were errors in the interpretation of initial images with all image processing techniques. This was particularly apparent from distortion, which usually occurs as a result of the lens geometry and due to diffraction in the glass box and the gelatine. The process of minimising the distortion and removing, to a reasonable degree, its influence on the data is described below.

Discrete speckles of cellulose-based paints were chosen for their stability, rapid solvent evaporation and organic nature. The speckles optimised the DIC output and did not provide another surface layer to the specimens. Uniaxial tests were performed on corneal tissue, both with and without applied media, to ensure no mechanical effects resulted from this process.

Referring back to the physical setup described in Fig. 1, the cameras were located orthogonal to the sides of the glass box. This minimises the distortion, which occurs as light was refracted while passing between the air and the glass and between the glass and the gelatine. The error in this system of measurement was quantified using the same setup as used to calibrate the laser beam. Multiple known positions within the gelatine were measured through DIC, and the resulting RMS error between known positions and their associated measurement was 1.7 μm.

**Fig. 3:**
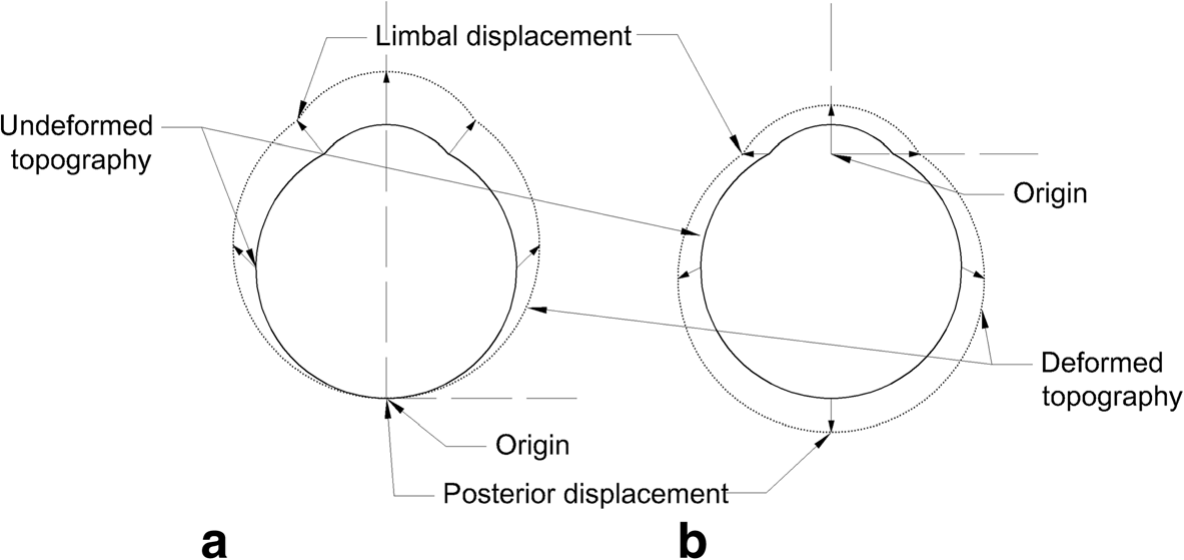
Diagrammatic representation of the analysis of displacement. **a** and **b** share the same reference and deformed topographies. **a** represents the displacement as initially measured. **b** shows the deformed image that has been reoriented such that the origin is the intersection point of the line representing the limbus with the axis of the laser beam

During the test, all deformations were measured relative to the single fixed position i.e., the junction with the inflation needle. However, if this measurement was used to perform inverse modelling, the deformation at the anterior of the eye would be a function of the deformation locally and the deformation of the posterior part, therefore, all modelling errors describing the response of the posterior region would be carried forward to the analysis of the anterior region. To rectify this, the position from which the relative displacement was measured was redefined. This process is diagrammatically described in Fig. 3; in (a) and (b) the non-deformed topographies are identical. In (b) the origin was moved to the intersection point of the line representing the limbus with the axis of the laser beam. Measurement of deformation was also obtained relative to the topography at intraocular pressure (IOP) = 2 mmHg, which was the minimum pressure necessary to remove initial wrinkles on the ocular surface. Ten discrete locations were measured from each camera: corneal apex and posterior pole; the mid cornea, limbus, equator and posterior sclera on the left and right sides of the images. In total, twenty-six measurements of deformation were obtained from each camera. The deformation at more points could be obtained using this process, but it was found in this work that having pressure-deformation behaviour at 26 points was sufficient for obtaining unique values for the material parameters of ocular tissue as described below.

### Geometric modelling

Accurate measurements of deformation and loading, and accurate representation of boundary conditions are required to derive representative material properties from an inverse analysis process. Additionally, the reliability of the derived material properties is reliant on an accurate representation of the geometry of the specimen within the finite element simulation. This section describes the method used to recreate the geometry of the specimen in the form of an FEM and the method used to quantify and minimise errors.

**Fig. 4:**
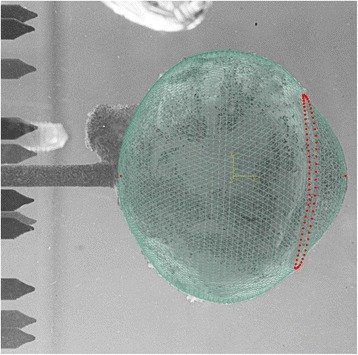
Match between modelled and imaged topography of the eye globe. The nodes representing the corneal apex, posterior pole and limbal ring are highlighted in red

The geometry of the models was constructed as an orphan mesh for use with Abaqus using bespoke software. The external topography was created from six individual meridian profiles from the three cameras at IOP = 2 mmHg. These profiles were calibrated and aligned with each other in the 3D space based on the common polar axis, which was defined by the needle. The internal topography was based on the external topography and eight meridian profiles of discrete thickness measurements. The thickness was measured at 2 mm intervals along each meridian from pole to pole [21]. The internal and external 3D topography was interpolated based on the relative spherical coordinates between the discrete points of measurement. The resulting FEM of the eye globe was free from restrictions of rotational symmetry. The images used to construct the geometry were calibrated by camera constants: the angle, the central position of the polar axis in the image, and the mm/pixel ratio which ranged from 0.0120 to 0.0122 for the three cameras (Fig. 4). The error in this method was determined by numerically reconstructing a Grade 100 Chromium (AISI52100) hardened ball bearing with diameter measurements of 12.5 mm ± 2.5 μm. The ball, which was contained within the gelatine, was numerically reconstructed with a resulting RMS error of 49 μm.

**Fig. 5:**
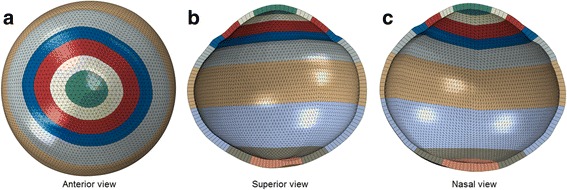
Finite element model of a tested porcine eye globe. (**a**) Image viewed from the corneal apex. Images (**b**) and (**c**) are cross-sections viewed from the equator. Image (**b**) sections through the reduced thickness location representing the lamina cribrosa. All images show the regions of various material definitions in the model, with dark blue representing the limbal region

The lamina cribrosa was represented by a thinned section of the FEM. The central thickness of this section is fifty percent less than the value in surrounding tissue; in addition the material was represented with a Young’s modulus of 0.32 MPa and a Poisson’s ratio of 0.49 [42]. Boundary conditions were provided to restrict the model from free body movement and provided an origin of deformation, which matched that applied to experimental data. IOP was represented by a distributed surface load (Abaqus keyword, *DSLOAD) applied to the internal surface of the model. This loading is appropriate in these cases where no external loads are applied and cavity volume output is not required. The models were constructed from seventy circumferential rings of quadratic, wedge-shaped, hybrid elements (Abaqus, C3D15H). The element type and the quantity have been chosen to create a smooth geometric representation, minimal volume locking, isotropic response to isotropic strain and adequate mesh refinement while keeping processing time at a reasonable level. The resulting model is shown in Fig. 5, which also shows the nine regions (colour coded) of the FEM, which provide nine ocular regions, each assumed to have unique material properties. The arrangement of the nine regions in the anterior-posterior direction was justified by the deformation maps obtained from the DIC analysis in which the deformation contour lines were mainly parallel to the coronal plane of the eye. The number of regions was determined to provide the greatest model refinement achievable, while remaining able to provide unique values of the material parameters for tissue in each of the nine segments within an inverse analysis process.

### Inverse analysis

Ocular material behaviour parameters were derived from the experimental data using HEEDS (v6.1, Red Cedar Inc., USA), in conjunction with Abaqus. The optimisation was based on determining material parameters that provided the best possible match between applied pressure and deformation results, as obtained experimentally and predicted numerically. The optimisation was based on the SHERPA algorithm that utilises Monte Carlo sampling to enhance the robustness and uniqueness of the solution. The algorithm’s objective function was to minimise the RMS error between the sets of experimental and numerical data as percentages of the final deformation at the relevant location;

1 $$ RM{S}_{\%}=\frac{1}{M}{\sum}_{j=1}^M\frac{\sqrt{\frac{1}{N}{\displaystyle {\sum}_{i=1}^N}{\left({\delta}_{i,j}^{experimental}-{\delta}_{i,j}^{numerical}\right)}^2}}{\delta_{max,j}^{experimental}}\times 100, $$

where *N* is the number of pressure levels, *M* = 26 is the number of measurement locations and *δ*_*i,j*_ is the deformation at each particular pressure, at each location. As the deformation was measured relative to the geometry at IOP = 2 mmHg, FEA was performed over two stages. The first stage elevated IOP to match that of the initial pressure of the experiment, and the second stage increased IOP to the final pressure. The deformation from the FEA (*δ*_*i*_^*numerical*^) was recorded relative to the beginning of the second stage.

The numerical model used to describe the material behaviour of the ocular tissue during loading was the hyperelastic Ogden model, utilised in a number of previous studies on soft tissue [43–45], and presented in Equation 2 in terms of the strain energy per unit volume, *W*:

2 $$ W={\sum}_{i=1}^N\frac{2{\mu}_i}{\alpha_i^2}\left({\lambda}_x^{\mathit{\hbox{'}}{\alpha}_i}+{\lambda}_y^{\mathit{\hbox{'}}{\alpha}_i}+{\lambda}_z^{\mathit{\hbox{'}}{\alpha}_i}-3\right)+{\displaystyle {\sum}_i^N\frac{1}{D_i}}{\left(J-1\right)}^{2_i} $$

where *λ*_*k*_^' *α*^ are the deviatoric principal stretches equal to *J*^− 1/3^ × *λ*_*k*_(*k* = *x*, *y*, *z*); *J* = *λ*_*x*_ ⋅ *λ*_*y*_ ⋅ *λ*_*z*_ where *λ*_*x*_, *λ*_*y*_, *λ*_*z*_ are the principal stretches in the three main Cartesian directions. The material parameters denoting the strain hardening exponent and the shear modulus are *α*_*i*_ and *μ*_*i*_(*i* = 1 … *N*) respectively, where *N* is the function order. The product of stretch in all three directions, *J* = *λ*_*x*_ ⋅ *λ*_*y*_ ⋅ *λ*_*z*_ = 1, following the approximation that ocular tissue is an almost incompressible material [46, 47]. The values of material parameters *α*_*i*_ and $\mu$_*i*_ represented the output of the inverse modelling process described above. The use of a first order material model, *N* = 1, reduced computation time during the modelling procedure by reducing the number of variables requiring optimisation and was found to produce stable results.

In addition to the utilisation of Monte Carlo sampling to enhance uniqueness, uniqueness was ensured by repeating the inverse analysis twelve times. For each run, the baseline and boundaries of the solution space where altered within sensible but exploratory limits, (0 < *p* < 4*s*), where *p* is the optimized parameter and *s* is the best fit parameter value.

### Experimental procedure

To illustrate the results of the test method, one human eye and one porcine eye were tested and the results analysed to produce estimates of material parameters for different regions of the eye globes. The human eye of a 69-year-old male with no known ocular diseases was obtained fresh from the Fondazione Banca degli Occhi del Veneto, Italy. The porcine eye was obtained within 6 h of slaughter. Both eye globes were stored in 6 % Dextran prior to the test. The aqueous and vitreous were removed through the needle and the intra-ocular void was filled with 6 % Dextran. Once contained within the gelatine and connected to the test rig, the IOP was altered by the control system described above. Both the human and porcine eyes were loaded and unloaded from 2 to 60 mmHg at a rate of 40 mmHg/min, with a rest period of 1 min following each of 10 loading cycles. The peak load of 60 mmHg, which was above the normal physiological range, was chosen to reach the higher IOP ranges associated with eye rubbing and tonometry.

## Results

### Human eye

**Fig. 6:**
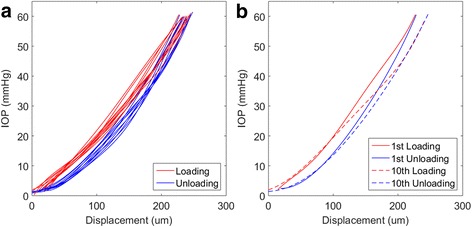
Load–displacement curves obtained for the human eye globe from the laser measurement device and pressure transducer. Displacement is measured in the coinciding axis of the laser and inflation needle. Plot (**a**) provides the curves for all 10 cycles including the recovery periods. Plot (**b**) highlights the 1st and 10th cycles

By providing a measurement of displacement from the posterior pole to the corneal apex with respect to IOP, the laser and pressure readings indicate the global response of the eyes. Figure 6(a) provides all displacement curves for the 10 cycles of loading applied to the human eye. This shows that up to 24 mmHg, there is no overlap between the loading and unloading curves of successive cycles. Above this level, there are still distinct trends within the displacement responses, which are independent of the cycle. Figure 6(b) highlights the first and last cycles where the difference in maximum displacement is 26 μm. Some differences can be found between the shapes in the displacement curves; between 24 – 45 mmHg, the global response of the human eye globe is more compliant in the last cycle. There is a reduced global viscoelastic effect evidenced when comparing loading and unloading curves for these cycles; this is particularly apparent at IOP >45 mmHg where the loading and unloading curves of the last cycle coincide. However, there is no significant difference in the curves up to 20 mmHg; both the first and last cycles exhibit similar relationships of global stiffness, non-linearity and viscoelastic effects. The recovery behaviour at 2 mmHg shows that there was complete recovery. The axial displacement observed at the beginning and the end of the tenth cycle indicates that there is less deformation than observed at the beginning of the test cycles.

**Fig. 7:**
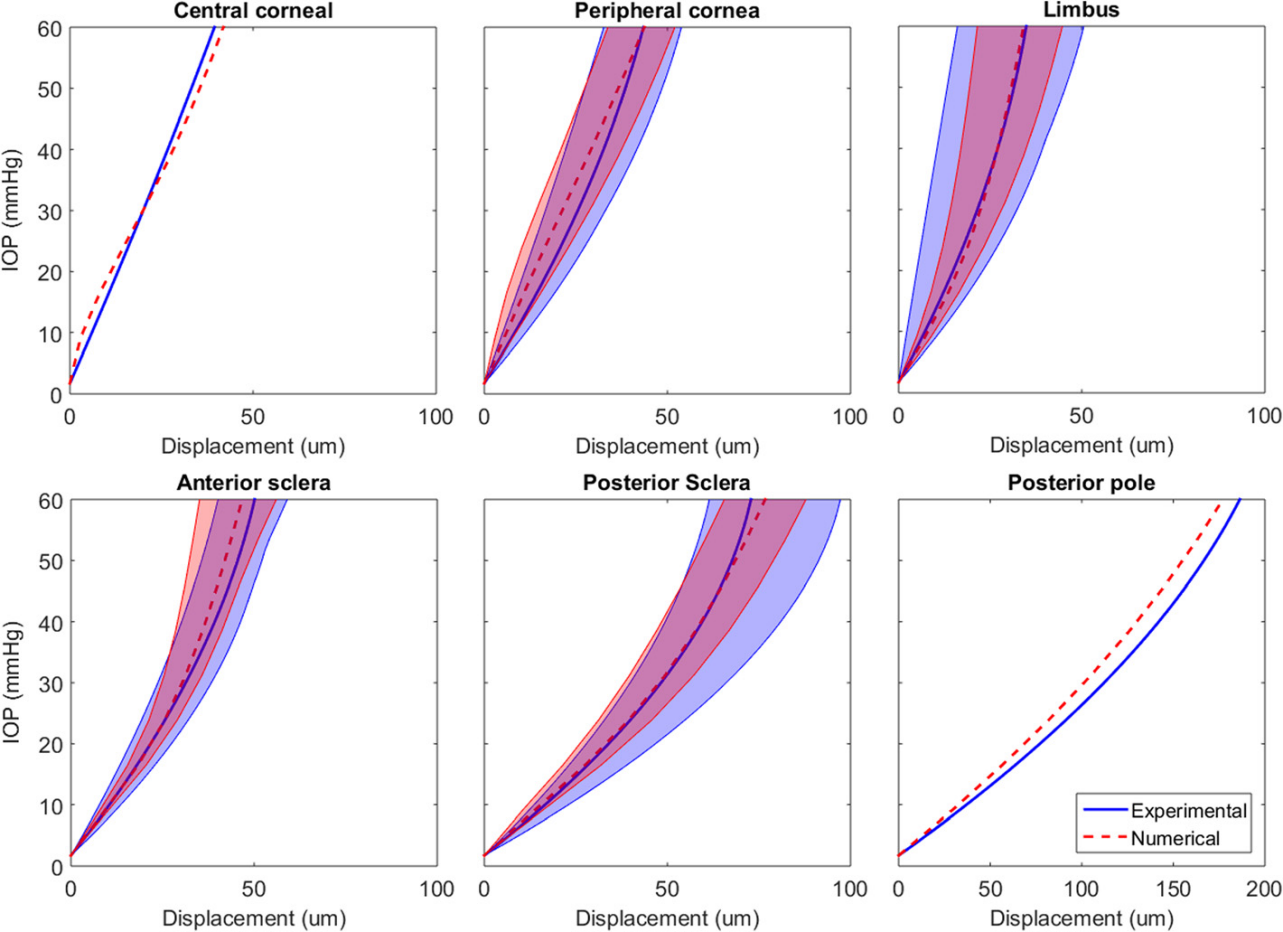
Experimental results obtained from DIC for the human eye globe with the corresponding numerical representation to 60 mmHg. Mean results for regional behaviour are represented by the thick dotted (*numerical*) and solid (*experimental*) lines. The shaded areas represent the range of results within the regions

As previously described, the DIC measurements of displacement have been reoriented. The resulting pressure-displacement curves for the human eye are provided in Fig. 7. The displacement measurement for the human eye in the central cornea has changed from 230 μm to 45 μm due the transfer of the origin from the posterior pole to the limbal region (see Fig. 3). The displacement of the posterior pole has increased from 0 to 185 μm in the same manner. However, the combined displacement along this axis measured by DIC remains consistent with the laser measurement, which was used for direct measurement and validation purposes. The peripheral cornea shows maximum displacement measurements from 33 to 55 μm at 60 mmHg with an average displacement of 44 μm. These measurements were obtained in different locations across the peripheral region and do not share the same polar or azimuth angles. Limbal displacement is now measured perpendicular to the axis of the laser and needle within a range of 15 to 46 μm with an average displacement of 31 μm. Similarly, displacements of 40, 30–48 μm (mean, range) and 62, 54–85 μm at 60 mmHg were measured in the anterior and posterior scleral regions respectively.

**Fig. 8:**
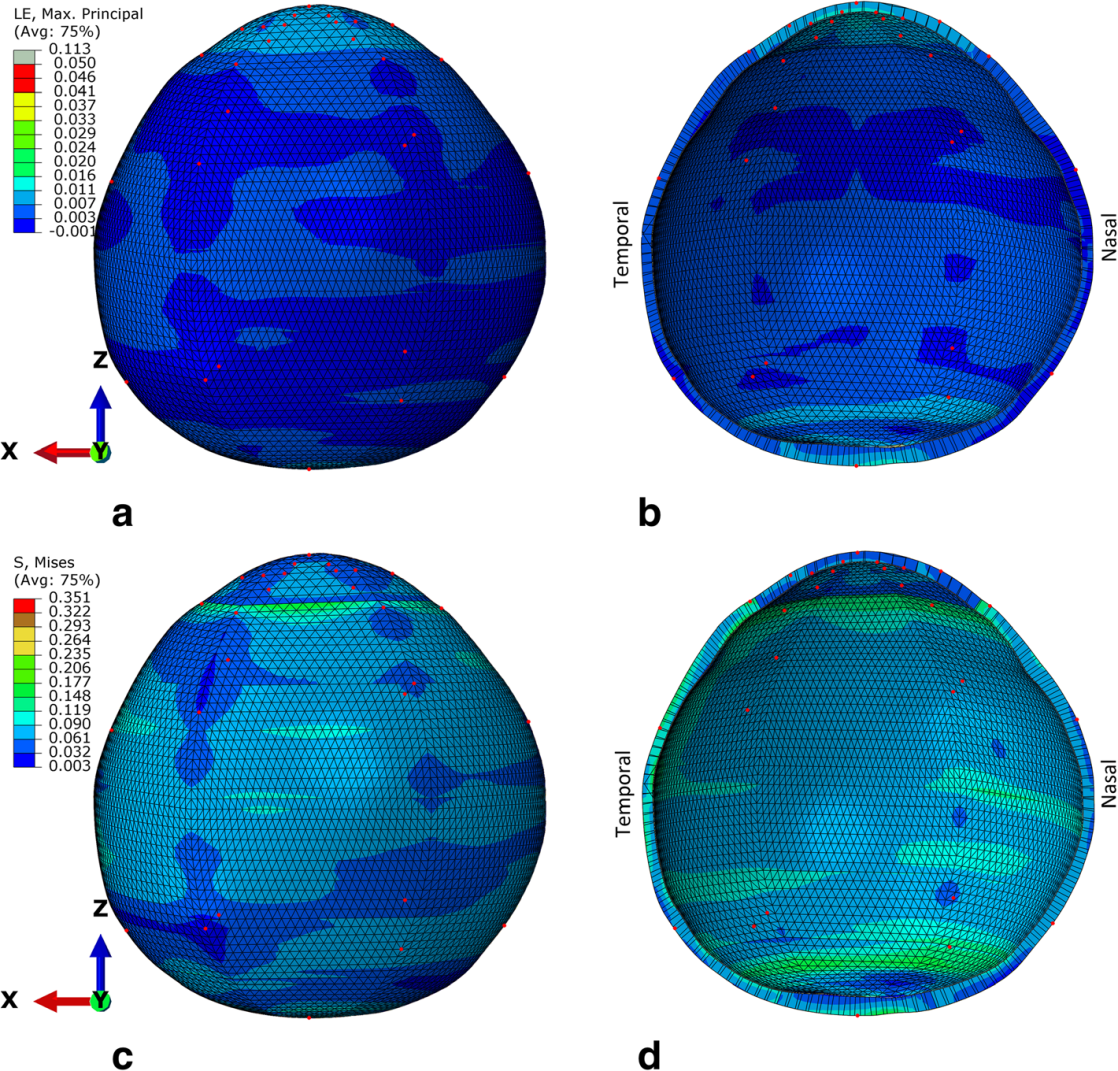
Finite element model of the human eye with stress and strain distribution plotted at 60 mmHg; as seen from the superior point-of-view. The z axis is collinear with the axis of the laser measurement and inflation needle during the test. **a** and **b** provide strain maps of the eye globe; while (**c**) and (**d**) provide the stress in MPa. **b** and **d** provide cross-sectional views that depict the variation of thickness and the lamina cribrosa. Red dots show the locations of the DIC measurements and fitting locations for the inverse analysis procedure

Inverse analysis has aimed to provide a match of the displacements measured from the experiment (Fig. 7) with those of the specimen-specific model shown in Fig. 8. An RMS error between the displacement predictions of the model and the experimental data of <10 % was achieved. Comparing the mean curves from within the peripheral cornea, limbus and anterior and posterior sclera regions, the RMS error was <8 %. The latitudinal variation in displacement response within these regions was small and has been represented most accurately in the peripheral cornea and anterior sclera. However, the latitudinal variation in displacement was larger, and hence less accurately represented around the limbus and in the posterior sclera, which contributed to the overall error in matching the experimental load–displacement behaviour.

The resulting material representations that have been derived for regions of the human eye globe can be considered by the numerical parameters *α* and *μ*, as seen in Table 2. *α*, relating to the non-linearity and stiffness, decreases from the central to peripheral cornea; is highest at the limbus; and reduces again towards the posterior pole region. *μ*, relating to the initial shear modulus, is lowest at the central corneal region; increases through the peripheral corneal to the limbus where again it is highest; and once again reduces from the limbal to posterior pole region. The uniqueness test showed that for seven out of the twelve inverse analysis runs, the best fit parameters varied by less than 0.5 % with similar RMS errors to the best fit of all the trials. The remaining five cases resulted in RMS errors ranging from 15-40 %. In these cases, the parameter boundaries were outside the range of the best fit solutions.

**Table 2:** Numerical parameters derived to represent the regional variation in material response for the human and porcine eye globes based on the Ogden model

Region	Human eye		Porcine eye	
	α	μ	α	μ
Central cornea	157.8	0.8100	220.1	0.0181
Peripheral cornea	117.1	1.332	114.3	0.0132
			110.2	0.0092
Limbal region	472.4	4.844	127.2	0.545
Anterior sclera	370.1	4.716	162.3	0.565
Equatorial region	344.9	5.920	181.3	0.642
	228.4	4.468	164.2	0.673
Posterior sclera	169.3	3.784	134.2	0.621
	157.6	2.672	118.2	0.592
Posterior pole region	127.9	1.894	95.62	0.535

Biomechanical representation can be considered by the resulting contour plots of stress and strain across the eye globe, as provided in Fig. 8 at 60 mmHg. In the model, the lamina cribrosa exhibits the greatest stress and strain due to its mechanical compliance and linear representation. Across the stroma, the cornea exhibits the greatest strain in the model. Unlike the derived material behavioural properties, there is no significant trend in the strain from anterior to posterior sclera. The stress in the cornea at IOP = 60 mmHg is similar to the majority of the sclera. However, there are areas of the sclera, which exhibit higher stress particularly in the temporal region. The region of highest stromal stress is in the limbus.

### Porcine eye

**Fig. 9:**
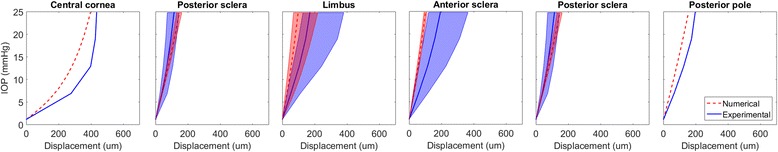
Experimental results obtained from DIC for the porcine eye globe with the corresponding numerical representation to 25 mmHg. Mean results for regional behaviour are represented by the thick dotted (*numerical*) and solid (*experimental*) lines. The shaded areas represent the range of results within the regions

DIC results (Fig. 9) obtained from the porcine eye reveal that the eye globe was relatively compliant at low IOP with highly nonlinear displacement responses; which was particularly apparent in the cornea. Experimental and numerical results are provided to 25 mmHg, above which the behaviour was essentially linear. At this IOP, the central cornea exhibits displacement of 400 μm. The peripheral cornea, limbus and anterior sclera exhibit large latitudinal variation in displacement within each region; this characteristic reduces towards the posterior pole.

The inverse analysis for the porcine eye provided material parameter estimations with a RMS error at 26 % due to the significantly larger latitudinal variations in displacement within each eye region. The values of material parameters, *α* and *μ*, obtained for the tested eye are presented in Table 2.

## Discussion

A significant benefit of intact globe testing is the ability to estimate the regional variation of stiffness across the cornea, limbus and sclera. Previous experimental analysis of these variations has mostly been limited to separated corneas and scleras, obtained from different donors in most cases. The limbal region is commonly used for clamping the separated cornea and sclera specimens and hence is not usually characterised. Additionally, the clamps provide unrealistic boundary conditions, which are likely to affect the behaviour obtained experimentally in the adjacent areas. Therefore, the procedure described in this study is of particular benefit for obtaining material stiffness properties at the corneoscleral junction, providing the eye with physiologic loading and supporting conditions and correlating the behaviour in the cornea to that in the sclera.

The main objective of this study was to provide a method for determining the material stiffness of the eye globe, which more accurately represents the in vivo state. The microstructural effects of tissue manipulation (as is inherent in strip uniaxial testing), and the associated deviation from in vivo strain distributions, have led in other test methods to the necessity for preconditioning before a consistent, cyclic stress–strain behaviour is observed [48]. The realignment of collagen fibrils during load cycles has been characterised by Quinn and Winkelstein [49] for ligaments, which also consist mainly of collagen fibrils. Quinn and Winkelstein’s study demonstrated a strong correlation between changes in collagen fibril alignment and changes in the mechanical response during preconditioning cycles as fibrils are able to change direction towards that of the principal strain. The pressure-displacement measurements of the eye globes tested suggest that no stiffening occurred between the 10 repetitive cycles, possibly as a result of no microstructural changes taking place. Subsequently, there is reduced need for pre-conditioning with the intact eye inflation procedure, which loads and supports the eye in a similar fashion to the in vivo conditions.

The limited experimental testing of one human and one porcine eye, included in this study, was presented mainly to illustrate the whole-globe inflation and the results that can be obtained from it. While the results presented show interesting trends that could only be obtained through whole-globe testing, they will need confirmation in studies involving larger numbers of test specimens.

In the test conducted on a human eye, inverse analysis allowed observation of high stiffness at the limbal region and the ring of scleral tissue immediately adjacent to it, reducing gradually towards the posterior pole. At the same time, the central cornea had a higher stiffness than the peripheral cornea, and both were much more compliant than the limbus. This behaviour is compatible with trends in material properties reported in previous studies [21, 27, 33]. While there was a decrease in the non-linear stiffness parameter, α, from the central to peripheral cornea, analysis revealed an increase in the initial shear modulus parameter, μ. This suggests a consistency with findings of previous studies which describe increased interweaving of collagen lamellae in the peripheral cornea [50]. On the other hand, while the derived material stiffness parameters reduce from anterior to posterior sclera, observation of the stress and strain maps reveal relative consistency across these regions – suggesting that these changes are compensated by the variation in thickness (geometric contribution to stiffness).

The inverse analysis included within the method description and performed in this study assumed isotropy of material behaviour. This is despite the fact that the mechanical response of ocular tissue is known to be anisotropic and that a number of studies have utilised characteristic anisotropic microstructure arrangements [51, 52] in numerical representation of the cornea [53, 54]. However, since there was no data providing characteristic microstructure relationships covering the entire eye globe, it was not possible in this study to adopt an anisotropic numerical representation of the tissue.

The results obtained immediately adjacent to the posterior pole may have been compromised by the insertion of the inflation needle. Due to the process of reorienting the DIC measurement such that the origin was no longer located at the posterior pole, the potential inaccuracies in this area did not compound smaller errors in the derivation of material parameters elsewhere across the eye globe. The response to increases in IOP is a global response and errors in the representation of one region will always affect another region. However, it is suggested that this transfer of the origin to the limbal region has improved the reliability in the derivation of properties particularly at the limbus and across the cornea as this effect has been limited.

For the porcine eye, while the experimental procedure was applied successfully, the inverse analysis produced larger errors due to considerable latitudinal variation in behaviour within each of the model’s nine regions. This is most prominent in the mid cornea, limbal and equatorial regions. While the non-rotational representation of the specimen specific geometry was considered, the FEM was limited to rotationally symmetric material representation, which meant that the variation in stiffness could only arise due to the slightly irregular geometry around latitudinal regions. However, it is clear from the fitting of numerical to experimental data in the porcine eye that variations in geometry alone did not entirely represent the variation in stiffness, suggesting the need to consider possibly significant variations in material stiffness around these latitudinal regions when modelling porcine eye globes. This makes it more important to consider the anisotropic fibril distribution across the ocular surface once this information becomes available.

## Conclusions

The study demonstrated that preconditioning cycles may not be required to obtain consistent load-deformation behaviour in globe inflation testing. This is a result of the test procedure providing a strain distribution that closely represents that of the in vivo eye and does not involve significant surgical manipulation as those factors would otherwise result in fibre re-orientation, and hence tissue stiffening, between successive preconditioning cycles. These findings are consistent with an earlier study on sections of non-human ocular tissue [55].

While the test procedure presented in this study represents a significant improvement in the ability to derive regional stiffness variations consistent with the in vivo eye, the method including the experimental testing and associated data analysis is substantially more demanding and more complex than uniaxial strip extensometry and inflation of separated corneas and scleras. However, despite these challenges and the difficulties in acquiring whole eye globes for research, the novelty of the technique and the more comprehensive data it offers, make the method invaluable in ocular material characterisation, especially as microstructure data covering the whole ocular surface becomes available.
